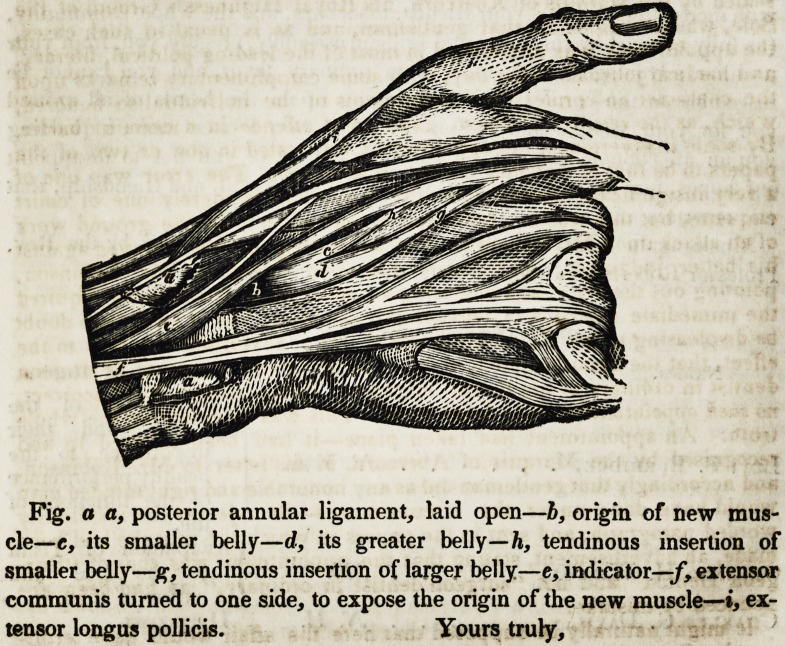# Miscellaneous Notices

**Published:** 1850-04

**Authors:** 


### Miscellaneous Notices.

***Tenth Annual Meeting of the American Society of Dental Surgeons, held in the city of Baltimore, the 25, 26, 27 days of March, 1850.***

The Society convened at the Lecture Hall of the Baltimore Dental College, on the 25th of March, 1850, at 12 M.

The first business transacted was the appointment of Committees, as follows:

1st. Committee to arrange the order of business.—Dr's A. Westcott, C. O. Cone, R. Arthur.

2d. Committee on grievances to examine and report upon papers pertaining to the moral standing of one of the members. On this Committee the president appointed—Drs. Cleaveland, Townsend, Foster. To which were added by nomination, E. Parmly, C. A. Harris.

The Recording Secretary here asked to be excused from keeping any minutes (for publication) relating to the business of the last named Committee, and moved that Dr. R. Arthur be substituted to keep the minutes pertaining to this subject. Dr. Arthur was so appointed. Adjourned till nine o'clock to-morrow.

BALTIMORE DENTAL COLLEGE, *March 26th,* 1850.

9 o'clock A. M.

The Society convened agreeably to adjournment.

The Committee on arrangements reported the following order:

1st. Reading by Dr. Harris, a Memoir of the late Dr. Nasmyth.
2d. Report of Committee on Grievances.
3d, " " Dental Education.
4th. " " Practical Dentistry.
5th. " " Tabular Sheet or Dental Record.
6th. " " Treasurer.
7th. " " Condition of the Journal.
8th. " " Discussions on Practical Subjects.

In pursuance of the above order, at ten o'clock, Dr. Harris read the Memoir of Dr. Nasmyth.

On motion of C. O. Cone the thanks of the Society were presented to Dr. H., for said Memoir and a copy requested for publication.

The reading of this paper elicited appropriate remarks from several members present, particularly from Dr. E. Parmly, after which, on motion of Dr. Foster, the Society tendered their thanks to Mrs. Nasmyth for presenting to the profession the result of the researches of her late husband.

The Committee on Grievances in the case referred to them reported progress.

Dr. R. Arthur from the Committee on Dental Education (appointed August, 1848,) reported that the duties of the Committee had not been fully understood, and hence nothing had been done. He therefore moved that the Committee be dismissed, and that the subject be divided and appropriate committees be appointed to take charge of the respective branches.

The old Committee was dismissed and Dr. Arthur was requested to name the divisions and to define them. Dr. A., then read a paper upon this subject, which gave rise to some discussion in which Drs. Arthur, Hullihen and several others participated. It was finally agreed that the division made should be into *Dental Literature* and confined to *American Literature* and Dental Education.

Persons appointed to prepare a paper, on the former, Dr. R. Arthur, on the latter, A. Westcott.

The Committee on Practical Dentistry was called, but none of its members being present, the subject was, on motion, laid over, and the former Committee, consisting of Dr. J. B. Rich, E. Maynard and Alexander Nelson, was continued.

Dr. E. Townsend here gave notice, that he wished at a proper time to present a subject to the Society on which a Committee might be called for. He wished also to present some other promiscuous business. It was therefore moved to suspend the regular order, pro tem.

Dr. T. then presented an application for membership from Dr. J. D. White, of Philadelphia.

He then after some introductory remarks, proceeded to read a paper which he had prepared for the Society. This paper was upon the subject of the "*Amalgam Pledge*" and after remarks from most of the members present, was, on motion of A. Westcott, seconded by Dr. J. H. Foster, referred to a Committee, to report upon the subject matter at the next meeting of the Society.

The Committee was appointed by the chair and is as follows:—A. Westcott, E. Townsend, J. H. Foster.

The Treasurer's books were received (the Treasuer E. J. Dunning, not being present) and a Committee was appointed to examine and report upon the same, as follows:—Drs. Harris, Cone and Arthur.

A Committee was also appointed to report at the next meeting upon the condition, prospects and future management of the Journal, as follows:—Drs. Harris, Westcott, Townsend, to which was added Drs. E. Parmly and Cone.

A resolution was offered by Dr. Foster, directing the Recording Secretary to enquire of the Corresponding Secretary, why he had so long retained the report of the last meeting (made by Mr. Burr, reported) thereby preventing the publication of the same, carried. Adjourned till to-morrow.

Baltimore Dental College, *Feb. 27th.* 10 o'clock A. M.

Dr. C. O. Cone made a verbal report on the "Tabular Sheet"—said it was not yet fully completed. On motion, he was requested to complete the same and report at next meeting.

The Committee on Grievances being called upon, the chairman stated that they should only be able during the present meeting to report progress in the case referred to them. Whereupon Dr. S. P. Hullihen, moved, that the report be accepted and the Committee discharged, and that a new one be formed, carried. On motion of Dr. Arthur, the same Committee was re-appointed with instructions to report at the next meeting.

Dr. Cone from the Committee on Journal, made a report which was ordered to be placed on file. Dr. Harris made a proposition to take a certain amount of the back numbers of our Journals towards the money he had advanced. His proposition was accepted and ordered to be placed on file.

On motion, Dr. Harris was authorized to collect dues for Journal and for membership.

Dr. L. S. Parmly was authorized to act as general agent for the Society, both in this country and in Europe.

Dr. W. H. Dwindle withdrew as an editor of the journal, and Dr. E. Maynard, of Washington City, was appointed in his place.

On motion of A. Westcott, a Committee was appointed to revise the Constitution, particularly that portion of it pertaining to the admission of members. The Committee is as follows:—Drs. Westcott, Hullihen, and Dunnings.

On motion it was resolved, that the officers of this Society, (except in cases of vacancy,) hold over till the next annual meeting.

Dr. S. P. Hullihen, moved that a Committee be appointed to prepare an address for the *public,* carried. Dr. S. P. Hullihen was appointed as such Committee.

*Discussions in Practical Dentistry.*—The subject of filling over exposed nerves without destroying them, was discussed at considerable length, Dr. Hullihen exhibited the kind of cap which he employed, and showed the manner of making and using it.

Dr. H., also exhibited a new method of putting teeth on to plate, also several ingenious fixtures used in regulating teeth.

Dr. Cleaveland exhibited a new method of constructing air chambers in plates, showing great ingenuity and skill both in design and execution. The general subject of cavity or air-chamber plates was very fully discussed.

Dr. E. Townsend exhibited fixtures for regulating teeth and explained their mode of application.

*Adjourned to meet on the second Tuesday of August,* 1850, *at Saratoga Springs.* A. Westcott, *Recording Secretary.*

---

*Annual Commencement of the Baltimore College of Dental Surgery. Session,* 1849–50.

The annual commencement for conferring the degree of Doctor of Dental Surgery, was held in the lecture room of the college on the evening of March 28th. The hall was at an early hour filled by an audience composed of ladies and gentlemen.

The examining committee composed of distinguished gentlemen both of the Dental and Medical Profession were all present, except three. It may be well to state that this committee was appointed upon the recommendation of the American Medical Association, whose duty it was to be present at the examinations of the Students. This committee consisted of S. P. Hullihen, M.D. of Wheeling Va.; E. Maynard, M.D. Washington, D. C.; J. H. Foster, New York; E. Townsend, D.D. S., Philadelphia; and S. Blandin, M.D., Columbia, S. C. with Dr. Eleazar Parmly, of New York, the Provost of the College, among dentists, while the medical portion of the committee consisted of Drs. J. J. Cohen, Dunbar and Wm. W. Handy, all of Baltimore. This committee, with three exceptions, were all present and took part in the examinations.

From the American Society of Dental Surgeons, which body held their annual convention at the same time in our city, were present both at the examinations and commencement, Drs. Westcott, New York; Cleaveland, S. C.; Levi Parmly, New Orleans; Gardette, Philadelphia; Dwinelle, New York; and Arthur, Washington City.

The Assembly thus constituted, the order of exercises was as follows:

1st. *Prayer* by the Rev. Dr. Holmes of Pittsburg. Then followed music.

After which the names, residence and subject of Thesis of the Graduating Class were announced by the Dean. Music again followed, and then the *authority* for conferring the degree was read, after which the graduates were called up by two's successively, to receive at the hands of the Provost, each a *diploma* as the highest honor, and testimonial of qualifications which could be bestowed.

After this, Dr. Townsend, of Philadelphia, gave a short and very appropriate address to the graduates, when—music again interposing—the *Valedictory* announced for the occasion was pronounced by Dr. S. P. Hullihen, of Virginia. It was replete with noble sentiments, burned with an enthusiasic love for the dignity, honor, and elevation of the dental profession, and claimed as the standard of its highest excellence, the whole broad basis of medical and moral science, as the necessary and only legitimate foundation to constitute an accomplished and scientific dentist. We cannot pretend to give in this place even so much as an outline of either of those productions, nor is it necessary, as they are both to be published, and we therefore defer any further remarks, and simply commend to the reader their attentive perusal.

To the above address Charles G. Davis, rose and delivered the following valedictory reply in behalf of the graduating class.

In behalf of the class whom I have the honor to represent at this time, I rise to return our most cordial thanks and deep-felt gratitude, to our much respected instructors, the gentlemen of the faculty.

Be assured, gentlemen, that we feel much more than we are able to express, the obligations under which we are laid, for those inestimable treasures of science which have been so freely, kindly and with such untiring assiduity unlocked to our research during the two years we have had the honor of listening to your instruction.

Nor can we feel that it is the *matter alone,* but the *manner* in which it was imparted, that has placed you all in our affections, and rendered pleasing every association connected with the halls of this institution. When in future years our minds recall the incidents of the past life, this college, its connections and associations will, I am confident, be one of the bright spots around which a halo of pleasing recollections will be thrown, inviting the memory to linger in dreams of delight, undisturbed by a single thought of one discordant note, occurring throughout our entire intercourse while here together.

And as we go forth from this institution under your auspices, protected by the *armor* with which you have invested us, directed by the principles you have taught us, we are not insensible to the emotions that must agitate your feelings as you follow us along the paths of our career, observing our efforts and various successes.

As the inventor looks on with solicitude, while the tests of his invention are in progress, and feels emotions of pride and pleasure at its triumph, so must the teacher who has prepared others for the active duties of life, watch with

*paternal* solicitude the progress of his pupils and feel a noble pride as he sees their efforts availing and themselves riding upon the tide of success.

And the humble hope of your class is, gentlemen, that based upon the foundation for acquiring knowledge which you have laid for them, having for their beacons of guidance unwavering principles of moral integrity, impelled onward in the paths of truthful research by a well directed zeal, having constantly the elevation of the profession in view, that if we can never boast of more we may so conduct our bark over the sea of professional life as to do no dishonor to our *alma mater,* or cause the veterans in our profession to blush at our names.

We extend our thanks to the gentlemen of the committee and others of our profession who have honored at this time by their presence, and interested and instructed us by their talents. And now with the kindliest recollections we leave you hoping for the smiles of a beneficent Providence to crown with ever increasing success, your noble and truly praiseworthy efforts for the advancement of science and the elevation of the standard of "professional excellence."

Dr. Davis having taken his seat, the Provost rose, and replied as follows:

Young Gentlemen, Graduates:

You have now received the highest distinction, and the highest honor, that the law of this State and the rules of this Institution authorize us to confer upon you. And in return for the assurance of the Faculty, which this Diploma conveys, we, in kindness and in confidence, ask you solemnly to promise that you will adhere to correct principles, both in your morals and in your practice; that in your social relations you will carefully and strictly avoid dissipation, dishonor, and disgrace; and in your practice you will avoid, with equal care, deception, imposition, and quackery:—and this you solemnly promise?—(to which they each responded yes.)

You have, my young friends, not only during your professional studies, but during your whole life, been accustomed to look towards the East for the dawn of light; but this evening it has broken in upon you, with greater than morning brilliance, from the West: and whether such truth comes to you from beyond the Rocky Mountains, or from the Valley of the Mississippi, value it as the richest gift which you can possibly receive, or which can be bestowed upon you.

There has been so much said to you, in eloquence and refinement, in truth and in sincerity, that it leaves nothing for me to add. I will therefore say but a few words, in reply to a remark or two contained in the exceedingly appropriate address just delivered in behalf of yourselves, in which you have so well expressed to your teachers the grateful feelings, which their kindness as friends, and faithfulness as instructors, has impressed upon you. In their behalf I would say, that the highest satisfaction that a teacher can feel, is in witnessing the advancement of his pupils, and in seeing in them the desire to attain to the excellence that is taught; and the highest satisfaction a pupil can feel is in seeing, day by day, that he is reaching that excellence, and with respect and affection to his teachers, feels the assurance that they are doing all they can do, for his future good; and from the proofs we have had of the past session, this has been mutually felt in no moderate degree.

You speak of the anxiety and solicitude an inventor feels in watching and witnessing the progress and success of his invention. Applying this to yourselves, it is an apt and happy illustration. The value of any and every invention, is the use to which it can be applied—not its form, its extent, or its finish—for however beautiful its form, however perfect its parts, or however polished its exterior, the first enquiry, by those who wish to employ or use it is, "Does it work well?" The three days examination we have just finished, and the very beautiful specimens of workmanship in mechanical science, that have been brought before the examiners, have given the highest proof that each one of you can work well; which important fact will be the highest commendation that you can receive to favor and success.

You have expressed the wish that you may be "able to guide safely your bark over the sea of professional life." Permit me to assure you, my young friends, that this sea is one that is extremely difficult to navigate. It is fraught with rocks, shoals, and quicksands, with calms, trade-winds, and tempests; it has its undertow, gulf-stream, and Cape Horn: and unless you are by far more successful than many of the older navigators have been, who are now before you and around you, you will, to use a sailor's phrase, (in the early part of your voyage at least,) often find "the wind dead ahead." The only way, then, for you to keep your bark steadily on her course, is to be accurate in your observation, careful in your reckoning, and after comparing your daily experience with your previous log, making truth and temperance your guide, and professional excellence the polar star at which you are aiming, towards which you are constantly steering, and at which you expect to arrive. I have said you must make temperance your guide; you must avoid then, as you would avoid death, the *stream* and the *Cape*, for no one ever yet reached professional excellence, that bright star of the North, by "running down the trade," or by seeing double, by too often "doubling the horn."

You have heard most eloquently and truthfully described, this evening, the wretched and degraded condition in which, a few years ago, our science lay; but a change has taken place in the minds of the community at large, who now look upon us differently, and regard us more favorably; and this is particularly the case among men of generous minds in the medical profession,* who regard this Institution with peculiar interest, standing as it

* This Institution is recognized by the American Medical Association, and its delegates received as members of that highly distinguished and honorable body—a distinction worthy only of being bestowed by the elevated minds and characters of those who control and sustain this pride of American Medical Institutions, based, as it is, in the noble brotherhood of kindred minds and kindred sciences, for the advancement and elevation of both.

does, solitary and alone, in the great field of science; and though ostentation and pride may regard it as holding no other than the same relation to the higher institutions of professional learning, than the smaller orbs of light do to the greater luminaries in the solar system: yet may they long exist, and move in harmony together, reflecting light upon each other, and however dimly or brightly that light may shine, may all who have received its beneficial influences, so diffuse it abroad, that every one who is benefitted at their hands, shall be fully persuaded that this and similar colleges are necessary to complete the number of those institutions of learning which have been established in our social system for the benefit of mankind.

The voice of admiration and approval of our efforts in the cause of science, has this day reached us from a noble spirit, belonging to a noble nation, beyond the sea. Who could hear the generous praise bestowed upon the American Society of Dental Surgeons, and upon the Baltimore College of Dental Surgery, in the brilliant address of Dr. Robinson, of London, (so well read to-day before the Society of the Alumni,) that would not feel proud of being a member of the one and a graduate of the other. Praise like this, from the most distinguished professional men of Europe, with such prospects as we have before us, cannot fail to encourage us to unwearied effort to endeavor to make our system of practice and system of learning worthy of imitation as well in distant ages as in distant lands. And wherever your lot may be cast, remember your obligations to perform well your part in this great work of perfecting dental science and dental practice. We look to you with increasing interest; your friends, associates, and the whole community, (as fully evinced by this large and brilliant assembly,) regard you, from your peculiar advantages, with more than ordinary favor, and you cannot be insensible to the earnest desires springing up in the hearts of all around you for your welfare and happiness. And may I once more, in bidding you an affectionate adieu, most earnestly entreat of you not to disappoint our hopes, nor forfeit the feelings of respect, good will, and friendship, that we entertain and cherish in your behalf. Farewell.

Music again following. The Benediction was pronounced by the Rev. Professor Roberts, of Baltimore, and the assembly dismissed.

*List of Graduates for Session,* 1849–50.

| Names. | Residence. | Subjects of Thesis. |
|---|---|---|
| Levi S. Burridge, . . . . . . . . | *N. York.* | Preservation of the Teeth and their importance to the healthy performance of the functions of life. |
| Orlando H. Wilcox, . . . . . . | *Md.* | Adaptation of Teeth to the food and nature of man. |
| Charles G. Davis, . . . . . . . . | *N. H.* | Dyspepsia. |

| Names. | Residence. | Subjects of Thesis. |
|---|---|---|
| J. Dickson Smith, M. D., . . . . . | *Ga.* | Philosophy of Plugging Teeth. |
| Seraphim H. Dumont, . . . . . . | *Belgium.* | Maxillary Sinus, its position, form and diseases. |
| Fendal D. Thurmand, . . . . | *Va.* | Odontolgia. |
| D. Lafayette Stocking, M. D., . | *La.* | Treatment of Dental Pulp or Exposed Nerves. |
| Henry B. Young, . . . . . . . | *Ohio.* | Advantages and disadvantages of the inclined plane to treatment of Dental irregularity. |
| Robert Johnston, . . . . . . . | *Va.* | Cause and effect of Caries of the Teeth. |

Baltimore, *April 22d,* 1850.

*My Dear Doctor:*—I observe that the explanation of the wood cut in the last number of your Journal, giving an account of a new muscle of the hand, does not correspond with the lettering upon the drawing. You will therefore, please introduce the drawing again in your next number, with the following corrected explanation of the plate.

Fig. *a a,* posterior annular ligament, laid open—*b,* origin of new muscle—*c,* its smaller belly—*d,* its greater belly—*h,* tendinous insertion of smaller belly—*g,* tendinous insertion of larger belly—*e,* indicator—*f,* extensor communis turned to one side, to expose the origin of the new muscle—*i,* extensor longus pollicis. Yours truly,

To Professor C. A. Harris. W. R. HANDY.

Court Patronage and Professional Jealousy in England.—*From an English Correspondent.*—The present communication will furnish the professional public of the United States with some rather curious revelations of the working of a system from which, we trust, they are exempt; indeed, we have reason to believe, that in no civilized country in the world but this, could so barefaced and unjustifiable an interference with the right of the government (whatever its form may be) to confer, or the claim of the individual to receive, the usual honors and distinctions to which he was fairly entitled, be permitted, without subjecting the party at once to the contempt and indignation of his professional brethren and the public. The following statement, for the accuracy of which we can vouch, will show the difficulties in which professional men are occasionally placed, and the nature of the opposition they have to contend against, when back-stairs influence, and the underhand working of a clique are resorted to for the purpose of monopolizing honors, and emoluments, and crushing, if practicable, all fair and honorable rivalry.

In the month of May last, Mr. James Robinson, of Gower street, who, if he does not hold the very highest position, enjoys, at least, one of the most extensive dental practices in the metropolis, having among his patients a large number of the aristocracy, including several of the immediate personal attendants upon her Majesty and the Prince Albert, was induced at the suggestion of several of the nobility to apply in the usual form for the honorary appointment of surgeon dentist to his Royal Highness. After some little delay, a warrant appointing Mr. Robinson "Surgeon Dentist to His Royal Highness Prince Albert," duly signed and sealed by the Marquis of Abercorn, his Royal Highness's Groom of the Sole, was forwarded to that gentleman, and as is usual in such cases, the appointment was announced in most of the leading political, literary, and medical journals of the day, with some complimentary remarks upon the character and professional attainments of the individual so honored, which, as the result will show, gave great offence in a certain quarter. By some inadvertence, the appointment was stated in one or two of the papers to be that of surgeon dentist *in ordinary.* The error was one of a very insignificant character, as the distinction is merely one of court etiquette, but this was the point seized hold of, to form the ground work of an attack upon Mr. Robinson. The Marquis of Abercorn, acting against his better judgment, immediately addressed a letter to Mr. Robinson, pointing out the error, and enclosing an advertisement which he required the immediate insertion of, *without any comment,* "as it would no doubt be displeasing to his Royal Highness." The advertisement was to the effect, that the announcement of Mr. Robinson's appointment as surgeon dentist in ordinary to his Royal Highness Prince Albert, was incorrect, *no such appointment having taken place.* This was true, but not the whole truth. An appointment had taken place—it had been referred to and recognised by the Marquis of Abercorn, in his letter to Mr. Robinson, and accordingly that gentleman did as any honorable and right minded man would have done under the circumstances—he rejected the insidiously worded paragraph, and sent to the papers in which the mistake had been made, an advertisement, stating that the appointment was that of "Surgeon Dentist" and not "surgeon dentist in ordinary," as had been announced by mistake.

It might naturally be supposed that here the affair would have terminated. Such, however, was far from being the case, and Mr. Robinson having declined to commit himself by publishing an indirect falsehood, a change of tactics became necessary. Two further communications were received by Mr. Robinson, from the Marquis of Abercorn, the purport of which were, that his Royal Highness had expressed his surprise at no *proper* contradiction having appeared, and then the ground was shifted. The publicity that was given to the appointment was objected to, as if a public announcement did not constitute the very nature and essence of all such honorary distinctions, and as if Mr. Robinson did not, in his application for the warrant, expressly state to the Marquis of Abercorn, that his object in wishing to have it immediately issued, was, in order to insert the honorary distinction in the title page of a new edition of his work on dental surgery, a copy of which he enclosed. The untenable nature of this objection must have struck his lordship at the time he made it, for, in the same breath, he hinted at the possibility of some informality in the issue of the warrant, made a suggestion as to its remaining in abeyance, and threw out a threat of its being withdrawn, summing up the whole with the somewhat inconsistent observation, that had Mr. Robinson inserted the insidiously worded paragraph above referred to, no further notice would have been taken of the matter.

Having failed in inducing Mr. Robinson to publish the contradiction so arbitrarily dictated, the Marquis of Abercorn condescended to do it himself. Immediately on its appearance in one of the daily journals, Mr. Robinson, by the advice of his friends, sent his warrant of appointment to the different journals for inspection, and they all from that period declined to insert the Marquis of Abercorn's advertisement, one of them "*The Daily News*" making the *amendé honorable* in its publication of the 8th August, in the following terms: "The paragraph which appeared in this paper, stating that no appointment whatever had been in contemplation to Mr. James Robinson is without foundation. The warrant of his appointment as Surgeon Dentist to His Royal Highness Prince Albert has been exhibited at this office." We now proceed to the third act of this singular drama in which a new actor appears upon the scene, and we obtain some insight into the machinery by whose agency in all probability much of what had gone before had been effected.

The Marquis of Abercorn finding that the public journals, after having seen Mr. Robinson's warrant of appointment, refused to insert his authorised and official contradiction, prudently retired from the arena. His place was, however, taken, by a party evidently not very scrupulous as to the means by which he might crush a rival. Accordingly, in the *Athenæum*, *Lancet*, and *Medical Times*, an advertisement appeared, of which the following is a copy: "Chesterfield House, August, 1849. This is to certify, that a warrant of appointment in the possession of Mr. Robinson, dentist, of Gower street, with my signature to it was given through inadvertence, and has been in consequence withdrawn, (signed) Abercorn." On the appearance of this extraordinary publication, Mr. Robinson never for a moment doubting that it had emanated from the Marquis of Abercorn, addressed to his lordship an indginant remonstrance, demanding to be set right with his professional brethren and the public. Pending the reply of his lordship, who was at the time at his shooting lodge in a remote part of Scotland, Mr. Robinson caused the following notice to be published in the *Morning Herald*, and several other newspapers: "A paragraph which was inserted in this paper on Tuesday last, relative to the appointment of Mr. Robinson, Surgeon Dentist, of Gower street, being calculated to mislead, we deem it right to state, that Mr. Robinson received the usual warrant of appointment as Surgeon Dentist to His Royal Highness Prince Albert, from the Marquis of Abercorn his Royal Highness's Groom of the Stole. It was announced by an error in some of the papers, that the appointment was that of Surgeon Dentist in ordinary, and an intimation was conveyed to Mr. Robinson that the announcement was, in this respect, informal; but not satisfied with a correction of the error 'in ordinary,' paragraphs were inserted in several of the papers, *upon what authority remains to be seen,* stating that the appointment itself was 'utterly unfounded,' and that it 'had never been even contemplated.' Mr. Robinson felt it due to his own character to explain the circumstances and exhibit his warrant of appointment to us as well as to other parties. The result of a lengthened correspondence has been an advertisement from the Marquis of Abercorn, stating that the appointment had been sent by him to Mr. Robinson 'through inadvertence,' and was 'thereby withdrawn.' The whole of the circumstances connected with this affair are so extraordinary, and the manner in which Mr. Robinson has been treated so uncourteous, that we understand, acting upon the advice of his friends, it his determination to lay the whole of the facts before the public."

Well might Mr. Robinson say that the circumstances were extraordinary, when the very next day brought him a letter from the Marquis of Abercorn, dated from Ardverekie Lodge, in Scotland, in which he states, "In answer to your letter of the 12th, in which you complain of a paragraph having been inserted in the newspapers, that a warrant had been given you through 'inadvertence,' I beg to state that I had no intention of having such a paragraph published, nor was I aware of its having appeared until informed by you," and further on, "I am at a loss to know by what mistake it could have been published." To prevent the possibility of any mistake, Mr. Robinson also wrote to the Solicitor of the Marquis of Abercorn, in London, and received the following reply:—"16, Clifford's Inn, 21st August, 1849. Sir, in answer to your inquiry whether the advertisement which has appeared in the *Athenæum* purporting to be signed by Lord Abercorn, relating to yourself, has been inserted by his lordship, or by me, as holding the original certificate, I have to inform you in addition to the assurance which his lordship has given you under his own hand, that such advertisement was not inserted by me, nor was I aware of the circumstance until I received a letter from his lordship, yesterday, on the subject. I am, sir, your obedient servant, John Froggart." After this extraordinary revelation, not having a very clear perception of where truth ended and fiction began, Mr. Robinson again addressed the noble lord's solicitor in the following terms: "7, Gower street, August 22. Dear sir, I am much obliged by your letter, stating that you had no knowledge of, nor did not sanction the advertisement in the *Athenæum*, signed 'Abercorn.' Will you also inform me whether the Marquis of Abercom, or yourself, as his agent, authorized in any way the insertion of the following advertisement in the *Daily News*, &c. 'We are desired, on authority, to state, that the report of Mr. James Robinson having been appointed Surgeon Dentist to his Royal Highness Prince Albert, is entirely without foundation, no such appointment ever having been in contemplation.' The same advertisement as appeared in the *Athenæum*, has been inserted in the *Lancet*, and *Medical Times*. Am I to understand that the Marquis of Abercorn and yourself are not in any way connected with their insertion, and that these advertisements have been inserted without his lordship's or your authority as his agent, as his letter implies? I shall feel obliged by your sending me a reply." The answer of Mr. Froggart was brief, lawyer-like, and decisive. "Dear sir, the only advertisements which have been inserted by direction of the Marquis of Abercorn, were those that appeared in the *Times* and *Morning Herald.* Your obedient servant, John Froggart." [This was the original paragraph which Mr. Robinson had refused to insert without comment.] Finding that some concealed and apparently unscrupulous enemy was at work to damage his character, Mr. Robinson directed his solicitor to apply to the newspapers in which these spurious advertisements appeared, for the name of the party who caused them to be inserted. The replies received from the *Daily News* and *Athenæum,* were simply a reference to Mr. John Froggart, solicitor to the Marquis of Abercorn, 16, Clifford's Inn! The trick was transparent enough—the parties inserting these advertisements not being aware that Mr. Robinson had been in communication with Mr. Froggart, flattered themselves that a reference to the solicitor of the Marquis of Abercorn would put a stop to all further inquiry on his part. That there might be no mistake, however, the answers from the *Daily News* and the *Athenæum* were forwarded to Mr. Froggart, and his answer was, that if he was referred to as the solicitor of these parties, "there must be some mistake in the matter—I have not the pleasure of being acquainted with either gentleman, and I have no instructions from them of any kind!" The deception that had been practiced upon Mr. Robinson and the public, was thus unmasked, and further evidence of authorship was not long wanting. The respectable publishers of Piccadilly, Messrs. Webster & Co., at the request of Mr. Robinson, had applied to the *Lancet* and *Medical Times,* with that object, and both these papers in the most honorable and creditable manner, at once admitted that the paragraphs signed "Abercorn" and dated "Chesterfield House" had been sent to them for insertion, by Messrs. Saunders & Ottley, publishers! When we state that the leading partner in the house of Saunders & Ottley is the near relative, we believe the father, of Mr. Edwin Saunders, who succeeded by *purchase* to the business and position of the late Mr. A. Nasmyth, Dentist to the Queen, we have said enough to show the origin and the animus of the discreditable persecution and attempted vilification to which Mr. Robinson has been subjected throughout this affair. Believing that it only required a full disclosure of such extraordinary proceedings, to induce the Marquis of Abercorn to take immediate steps to call to account the parties who had made so unwarrantable a use of his name, a full statement of the entire case was forwarded to his lordship, by Mr. Robinson. In justice to that gentleman we cannot withhold a few of his closing remarks. "Permit me, in conclusion, to observe, that highly as I should value an honorary distinction conferred upon me by any member of the royal family, its bestowal, or subsequent withdrawal without any good cause assigned, cannot add to, or detract from, my professional standing, so long as I can vindicate myself from the unjustifiable attacks which private jealousy or professional rivalry may dictate, and this, with God's blessing, I am resolved to do in the present, as in every other case." Having waited a fortnight to ascertain if his lordship would take any steps in the matter, Mr. Robinson resolved to lay a statement of the whole case before his Royal Highness the Prince Albert. Royalty, is said in this country to be "the fountain of honor," and the well known gentlemanly feeling, and nice sense of honor of His Royal Highness, would, it was anticipated, induce him to direct an immediate investigation. So far as any public intimation has been given, Mr. Robinson's expectations have, in this respect, been disappointed. The replies of the Prince, for more than one communication took place, are characterised by the courtesy and kindness that distinguish him. They contain repeated assurances that no slight was intended, nor no reflections could be cast upon his character, either professional or personal—the inadvertence or informality in the issue of the warrant is referred to, *but* not the slightest allusion or reference is made to the very extraordinary conduct of Mr. Edwin Saunders, or his relative. It was a matter upon which the Prince evidently did not dare trust himself to write—it would be too much, however, to infer from His Royal Highness's silence that he did not feel acutely the false position in which professional jealousy had placed the Court Dentist, and that he did not privately intimate his opinion of such conduct.

Such is a brief outline of this extraordinary affair which we believe is wholly unprecedented in the annals of dental practice or royal patronage. We should be glad to know if court appointments in England, are disposable to the highest bidder, like the pocket boroughs under the corrupt system that prevailed previously to the passing of the reform bill? If this be so, we should be curious further to ascertain the sum usually paid for the presentation, or whether an annuity is stipulated for in the shape of a percentage upon all fees to the party procuring the appointment, a subject upon which we shall have some curious and not very creditable revelations to make on a future occasion. So far as Mr. Robinson is concerned, we can vouch for it, having seen the whole of the correspondence, that he has not lent himself to any disgraceful system of bribery, fawning, or unworthy subserviency, in order to obtain or secure any appointment of the kind. It will scarce be credited that the name of a nobleman was surreptitiously and unwarrantably made use of for private and personal purposes—that after all the subterfuges which had been resorted to had been disclosed, and the trick discovered, neither the nobleman so libelled, nor the royal patron of the professional gentleman, both of whom were put in possession of the entire facts, took any steps so far as Mr. Robinson or the public are aware of, to mark their sense of such conduct. There is, however, one other tribunal, the court of last appeal—public opinion, and before the bar of that court, Mr. Edwin Saunders, or his relative now stands. The attempt to assail a professional rival covertly, and under the signature of another party, has availed nothing. The profession will, we feel, treat the case with somewhat less of courtly etiquette and delicacy, than it has hitherto experienced, and will expect the most ample and satisfactory explanation of all the circumstances. We have discharged our duty in making the facts public—it is for them as a body to vindicate the honor and the character of its members. To their hands we commit the issue, feeling satisfied that whatever course they may adopt, and whatever decision they may arrive at, will be such as becomes upright, intelligent, and honorable men. P. M. F.

*On the use of Chloroform in Dental Surgery.* By J. Chitty Clendon Higbey, Fleet Street, page 20.

This pamphlet is the production of the dentist to the Westminster Hospital. It contains his own experience in the employment of this anœsthetic agent for dental surgery. His statement respecting the difficulties in operating whilst a patient is under the influence of chloroform, appears to have been very great, more so than we have ever heard expressed by others, arising probably from a want of experience in the practice and administering of the agent itself. The pamphlet contains no new facts, it is extremely well written and printed. R.

Dr. C. A. Harris:

Enclosed I send you a small instrument which I invented and made for the purpose of measuring the depth of the hole in the root prepared or in preparation for a pivoted crown.

I have heretofore been in the habit of measuring with a small piece of wood, making a mark with an instrument at the base of the fang, but as I found this attended with some inconvenience I have made this instrument for that purpose. Perhaps you have something of the kind already, if so you will receive this for the motive by which it is sent, which is a motive that I think should actuate every member of the profession, viz: to add their mite for the benefit of the profession.

If you think it worth your acceptance, I shall consider myself well paid for sending it.

The manner of using it you will probably perceive at once. I place the slide near the end of the wire and introduce it to the fang, pushing up as far as it will go. The slide being larger than the hole in the fang is moved on the wire and thereby the depth of the hole is indicated. If the hole is found to be of sufficient depth, the pivot can be cut to correspond with that part of the wire projecting from the slide. Yours truly,

JEREMIAH MASON.

*Speculum Oris.*—We are indebted to Dr. W. H. Hull, for a beautiful speculum oris and tongue holder, invented by himself and manufactured by Mr. Jackson, a cutler and instrument maker of Baltimore. It consists of an elastic steel band so contrived that its size may be increased or diminished to suit the size of the mouth, with a plate about an inch and a half in length, extending from the lower side, which, when the instrument is placed in the mouth, depresses the tongue and prevents it from being moved. This instrument will be found particularly valuable in operating on the tonsils, fauces and uvula.—*Balt. Ed.*

*The American Medical Formulary.* By John J. Reese, M. D. Lindsay and Blakiston, Philadelphia, 1850.

This is a very convenient manual for the student of medicine and pharmacy, presenting, in a space as brief as possible, a complete list of the medicinal preparations now in use in England and the United States. The Appendix contains a good selection of recipes of Dietetic preparations; a Chapter on Poisons and their Antidotes; a table of doses, weights and measures, &c.—comprising a large body of most valuable information. We recommend it to our readers.

*Greencastle, April 4th,* 1850.

PROF. HARRIS:

*Sir:*—Enclosed is a tooth I extracted a short time since, which, upon examination, I discover to be injected with red blood. It is the first specimen of the kind that has ever come under my observation, during a practice of ten years, and if you deem it worthy of notice, I shall be happy in having contributed slightly in expelling any doubt of the vascularity of *human teeth.* C. VON BONNHORST.

---

*Researches on the Development, Structure and Diseases of the Teeth.* By ALEXANDER NASMYTH, F. L. S., F. G. S., member of the Royal College of Surgeons, London,—Fellow of the Royal Medical and Chirurgical Society. Pages 226, 8vo., JOHN CHURCHILL, London, 1849.

This is the title of a work recently presented to the medical profession, by the wife of the late lamented and distinguished author. We are informed that he had just completed the manuscript as *death* seized him.

The work throughout carries the impression of great carefulness, to arrive as far as possible at the truth, and an ardent desire for the elevation, dignity and more enlarged practical usefulness of the dental profession—a profession which was the unceasing pride and solicitude of all his thoughts.

This work of Dr. Nasmyth's comprises eleven chapters, having forty figures interspersed throughout, besides ten plates at the end of the volume, containing forty-one more figures, in all eighty-one illustrations, having a more or less direct bearing upon the teeth, most especially of their microscopical structure. The first chapter gives "the general physiology of the dental system." The second "descriptive anatomy of the mouth and jaws." The third and fourth, the description and minute anatomy of the teeth. The fifth "development of the formative organs of the teeth." The sixth "general and minute anatomy of the dental capsule and pulp." The seventh "development of the permanent teeth." The eighth and ninth, "the teeth an indication of an age, and progressive improvement of the human race." The tenth and eleventh chapters are devoted to the "development of ivory, and the chemical composition of the teeth." The plates generally are fairly executed, and especially those showing the vascularity and nerves of the dental pulp, and the vascularity of the mucous membrane of the tongue, gums, mouth, nose and palate.

These microscopical drawings seem all to have been gotten up without any reference to expense, but it would seem the main design was to combine as far as possible the three great requisites, viz: *accuracy, clearness* and *beauty* in their execution. . . . . . . .

*Dr. Solyman Brown's Circular.*—It would seem from a circular addressed to the members of the dental profession both in America and Europe, that our old friend, Dr. S. Brown, has opened an extensive establishment at 251 Broadway, New York, for the sale of S. W. Stockton's mineral teeth, as well as gold foil, plate, wire springs, solder, and in short every thing used by the dentist in the practice of his profession. It is also the intention of Dr. B. to keep dental works, magazines and other literary productions, proper for constituting and furnishing dental libraries.

From Dr. B's extensive acquaintance with the members of the profession as well as his knowledge of its wants, we know of no one better calculated to conduct the business in which he has engaged than he is. We take great pleasure, therefore, in commending his establishment to the attention and patronage of the profession.

As this is a matter in which the members of the profession generally are interested, we would publish his circular, but we are prevented from doing so for want of room. It can be had, however, by application to Dr. B.

---

*New Medical Journal Contemplated.*—We received a few days since, a prospectus of a new publication, to be entitled "The Baltimore Medical and Surgical Journal, the first number to be issued the first of July, but in consequence of the ill health of Dr. Hull, the editor and proprietor, it will be delayed, as we have been informed, until the early part of the fall. It is to be published monthly, each number to contain sixty 8vo. pages, at $3 per annum, payable in advance. We wish Dr. H. success in his contemplated enterprise. A medical journal, ably conducted, published in Baltimore, ought to be well supported.

---

*Omitted Articles.*—We are compelled, for want of room, to omit several articles and notices designed for the present Number. These shall appear in our next.—*Balto. Ed.*

## Figures and Tables

**Fig. f1:**